# Whole-genome sequencing in newborn screening? A statement on the continued importance of targeted approaches in newborn screening programmes

**DOI:** 10.1038/ejhg.2014.289

**Published:** 2015-01-28

**Authors:** Heidi Carmen Howard, Bartha Maria Knoppers, Martina C Cornel, Ellen Wright Clayton, Karine Sénécal, Pascal Borry, endorsed by the European Society of Human Genetics; the P3G International Paediatric Platform; the Human Genome Organisation; and the PHG Foundation

**Affiliations:** 1 Centre for Research Ethics and Bioethics, Uppsala University, Uppsala, Sweden; 2 Department of Human Genetics, Centre of Genomics and Policy, McGill University, Montreal, Quebec, Canada; 3 Department of Clinical Genetics and EMGO Institute for Health and Care Research, VU University Medical Center, Amsterdam, The Netherlands; 4 Center for Biomedical Ethics and Society, Vanderbilt University, Nashville, TN, USA; 5 Department of Public Health and Primary Care, University of Leuven, Leuven, Belgium

## Abstract

The advent and refinement of sequencing technologies has resulted in a decrease in both the cost and time needed to generate data on the entire sequence of the human genome. This has increased the accessibility of using whole-genome sequencing and whole-exome sequencing approaches for analysis in both the research and clinical contexts. The expectation is that more services based on these and other high-throughput technologies will become available to patients and the wider population. Some authors predict that sequencing will be performed once in a lifetime, namely, shortly after birth. The Public and Professional Policy Committee of the European Society of Human Genetics, the Human Genome Organisation Committee on Ethics, Law and Society, the PHG Foundation and the P3G International Paediatric Platform address herein the important issues and challenges surrounding the potential use of sequencing technologies in publicly funded newborn screening (NBS) programmes. This statement presents the relevant issues and culminates in a set of recommendations to help inform and guide scientists and clinicians, as well as policy makers regarding the necessary considerations for the use of genome sequencing technologies and approaches in NBS programmes. The primary objective of NBS should be the targeted analysis and identification of gene variants conferring a high risk of preventable or treatable conditions, for which treatment has to start in the newborn period or in early childhood.

## Background

### Next-generation sequencing technologies and genome sequencing approaches: the reality and the potential

The development of next-generation sequencing (NGS) technologies (ie, new high-throughput and massively parallel DNA sequencing technologies) has substantially reduced both the cost and the time required to sequence an entire human genome. The prospect of the availability of NGS technologies and consequently the greater facility to conduct whole-genome sequencing (WGS) have led some to predict that the use of this technology will change the current practice of medicine and public health by enabling more accurate, sophisticated and cost-effective genetic testing.^1^ It is foreseen that in the short term, the implementation of WGS in the clinic will improve diagnosis and management of some disorders with a strong heritable component,^2^ as well as improve personalized diagnosis and personalized drug therapy and treatment. Presently, NGS is being used for targeted sequencing of sets of genes to help guide cancer treatment, and a number of cancer centers are considering using WGS or whole-exome sequencing (WES) in the future. During pregnancy, noninvasive prenatal testing for aneuploidy is also being done using NGS.^3^ In the clinic, WGS and WES are also being used to identify the causes of rare genetic diseases especially in children^4^ and in individuals with ‘atypical manifestations, (that) are difficult to confirm using clinical or laboratory criteria alone, or otherwise require extensive or costly evaluation'.^5^ Disorders for which WGS has been used as a diagnostic tool are usually genetically heterogeneous and have variable phenotypic expression such as intellectual disability, congenital malformations and mitochondrial dysfunctions.^5^ Other foreseen applications include tissue matching, disease risk predictions, reproductive risk information, forensics or even recreational genomic information (such as genealogy or nonmedically related traits). Nonetheless, Goldenberg and Sharp^6^ predict that ‘it is likely that the earliest applications of whole-genome sequencing will be restricted to settings in which genetic testing is already a routine part of clinical or public health practice, such as state newborn screening (NBS) programs'.^6^ In truth, it should be noted that DNA testing, *per se*, is not a routine part of NBS and that only a very small proportion of babies, depending on the country, have a DNA test (as opposed to a biochemical test).^7, 8, 9^ Furthermore, the above prediction could be criticized as the routine nature of NBS with its often implied consent, together with its public health context, and the particular vulnerability of the population tested, would make it an unsuitable context into which to first welcome a WGS approach.

An important fact to keep in mind when discussing this topic and reading through this document is that NGS and WGS are not synonymous terms. NGS technologies are tools that can be used to sequence DNA (and consequently analyze sequence variants), as well as to allow for the study of RNA and epigenetic phenomena. In this article, we address the DNA sequencing capacity of NGS and subsequent analysis. Furthermore, the use of such powerful high-throughput sequencing technologies (whether they are the present ‘next/second generation' or any future similarly functional version, ie, third generation) does not dictate the amount of DNA to be sequenced or how much of it will consequently be analyzed. Admittedly, the sequencing approach can be determined by each researcher or clinician. For instance, the entire genome or the entire exome (protein coding regions) can be sequenced or one could decide to only sequence targeted regions or genes of interest. Another important point is that sequencing the entire genome or exome does not necessarily mean that every single variant will ultimately be analyzed; one can sequence the entire genome and then opt for targeted analyses of only certain areas or genes. Herein, we use the general term ‘genome sequencing' to include any high-throughput sequencing approach that offers the capacity to sequence large amounts of DNA without specifying how much DNA is sequenced or analyzed.

The goal of this statement is to analyze the relevant issues specifically surrounding the potential use of genome sequencing in publicly funded NBS programmes. The document culminates in a set of recommendations to help inform and guide scientists and clinicians, as well as policy makers regarding the necessary considerations for the use of genome sequencing technologies in newborns.

This paper is the result of a collaboration between the members of the Public and Professional Policy Committee of the European Society of Human Genetics (ESHG), the Human Genome Organisation Committee on Ethics, Law and Society, the PHG Foundation and the P3G International Paediatric Platform. The first draft was written and discussed by an editorial committee with representatives of the various groups. The paper and recommendations were discussed in the various groups and were posted on the ESHG website from 18 February 2014 until 13 March 2014 for public consultation. The final version of the paper and recommendations were approved by the ESHG Board and by the PHG Foundation in May 2014, and by the HUGO Committee and the P3G International Paediatric Platform in April 2014. The International Society for Neonatal Screening supports the consideration of all issues raised in this paper and recommends that it be used in guiding future debate on mutational analysis in NBS.

### NBS and its expansion

NBS is a public health program aimed at the early identification in asymptomatic newborns of conditions for which early and timely interventions can lead to the elimination or reduction of associated mortality, morbidity and disabilities. A test performed within a screening program is not intended to be diagnostic; rather it aims to identify individuals who are at sufficient risk to benefit from a referral for diagnostic testing.^10^ Traditionally, only a few rare disorders (eg, phenylketonuria or congenital hypothyroidism) were included in the screening program. Based on the Wilson and Jungner^11^ criteria, conventional neonatal screening programmes were limited to conditions considered an important health problem, whose natural history was well understood, required immediate medical intervention in order to prevent serious and permanent illness, and for which treatment was available. Based on this premise, NBS is usually conducted without an explicit consent because NBS is seen to be in the best interest of the child's health and as a consequence is considered as part of the routine care for newborns.

In recent years, the number of disorders offered on NBS panels has increased in both North America and Europe.^8, 9, 12^ At present, NBS programmes in the European Union (EU) are heterogeneous and aim to identify between 1 and 30 treatable conditions.^8^ The diversity is large; differences in the health-care systems' structure, available funds, local politics, input from professional groups, parent groups and the general public has led to different approaches in the way screening programmes have been set up, financed and governed. Confirmatory diagnostics and follow-up show large discrepancies. DNA testing is integrated as a final step in some programmes for cystic fibrosis screening and usually less expensive tests (esp., tandem mass spectrometry) are used first.

Canada has no national strategy on NBS and there is a wide variation between provincial programmes, including the number of diseases for which screening is offered, the information given to parents, consent and policies regarding the disclosure of carrier status.^13, 14^ In the United States, the Discretionary Advisory Committee on Heritable Disorders in Newborns and Children currently recommends 57 conditions for screening, including 31 core disorders and 26 secondary disorders,^15^ not all of which, as we briefly discuss below, adhere to the classic Wilson and Jungner^11^ criteria. This evolution has recently spurred controversy, with some critics arguing that the proposed expansion of NBS is proceeding too rapidly and without sufficient deliberation and care.^16^ It has been criticized for failing to ‘conform to contemporary standards of evidence-based decision-making'.^12^ It has been advanced that the panel includes conditions ‘that do not urgently need treatment in the newborn period, or for which no proven treatment is available, or for which the treatment is much less significant and certain than the benefit of treatment for a condition such as PKU'.^17^ This transformation has sparked debates on the objectives and rationale of NBS, as well as the associated evaluative and decision-making criteria.^16, 18, 19^

### NBS and genome sequencing

In line with Goldenberg and Sharp's^6^ prediction mentioned earlier,^6^ it appears to be a common expectation that once sequencing technologies are sufficiently robust and affordable, all newborns will have (at least part of) their genomes sequenced at birth replacing current tandem mass spectrometry used in NBS and potentially replacing any additional genetic test that could be needed later in life. For a number of reasons, this is not necessarily the best approach. As mentioned in a recent European expert opinion document, the first condition the authors recommend to be included in a NBS program is congenital hypothyroidism.^20^ This is usually not a genetic condition and cannot be diagnosed by genome sequencing. Therefore, for this condition, the present NBS methods cannot be replaced by sequencing.^21^

Predictions have been made suggesting that the implementation of sequencing technologies in newborns will become routine within a decade, affecting future generations of newborns. A vision of how newborns could be profiled was described over a decade ago in a UK White Paper on genetics. The authors suggested that it might be possible ‘to screen babies at birth' and ‘to produce a comprehensive map of their key genetic markers, or even their entire genome'.^22^ In this way, ‘the baby's genetic information could then be securely stored on their electronic patient record for future use. It could then be used throughout their lifetime to tailor prevention and treatment regimes to their needs as further knowledge becomes available about how our genes affect our risk of disease and our response to medicines'.^22^ Although there are presently practical, financial and ethical challenges that make this vision difficult to achieve or even unlikely for some, others, including Collins,^23^ believe that this is an inevitable end point in the development of personalized medicine, based on the argument that such screening would allow the continuous integration of genetic knowledge during the full length of our lives.

Importantly, it is also predicted that with the use of NGS, a greater number of genetic variants causing pediatric diseases can be detected ‘without substantially increasing the costs of NBS'.^6^ When considering NBS in general and more specifically the introduction of new technologies and/or approaches, the issue of costs is not trivial. Presently, the reported cost of the NBS procedure in 2011 in the EU ranged from € 0.46 per newborn (Serbia; screening for two conditions) to € 43.24 (the Netherlands; screening for 17 conditions),^20^ while sequencing and analyzing the data is much more expensive. Even when considering that Illumina recently announced that it could finally deliver on producing the $1000 genome,^24^ this price tag does not include the cost of managing or analyzing the data or of the health-care professionals' time to counsel and/or return results. Admittedly, at the moment, evidence of the economic impact of using WGS or other genome sequencing approaches in any context is lacking, and the real costs of the entire process including storage of data, data management and analysis, and return of results are, as of yet, unknown.^25^ In fact, the general agreement that dealing with the deluge of data generated from WGS or WES is far from an easy or obvious task, has led some to specify ‘the $1000 genome' and ‘the $100 000 analysis'.^26^ Therefore, the use of WGS or WES in NBS is not likely to fit within the available public health-care budgets at present.

Furthermore, the interpretation of DNA data in a population of healthy newborns is a challenge. The genotype–phenotype relationship in metabolic conditions is often not straightforward. In the case of Pompe disease, for instance, there is a large clinical diversity among patients with the same genotype.^27^ Furthermore, the sensitivity of sequencing analysis for specific disorders in each target population should be carefully considered, as it may be lower compared with present metabolic testing for some disorders. For instance, the current screening strategy for cystic fibrosis is deemed to have a sensitivity of over 95%. As not all disease-causing CFTR variants are known, the genotype first approach might have a lower sensitivity, with a wide variance in different populations. Also, the sequencing first approach would identify not only affected children but also the carriers of a combination of variants that might never cause a significant disease, or for which genotype–phenotype correlations are lacking or unsatisfactory.^21^ In this situation, there would be a concrete risk that these children would be, nevertheless, considered ‘affected' with the potential consequences of overtreatment, as well as alterations in family dynamics. An agenda for the responsible translation of genome sequencing in NBS would include determining the prognostic value of the variants identified.

A separate and equally important issue is the storage of large amounts, potentially the entire genome's worth, of sequence data. One could argue that the genome of a newborn could be sequenced once, and analysis later in life could focus on disorders relevant at that (future) age. Storage of genetic information, however, raises a host of questions, ranging from governance and privacy protection to ensuring the stability and accessibility of the data.^28, 29^ Furthermore, there is the very real possibility that newer sequencing technologies that can provide more complete and more accurate data more inexpensively will emerge. Thus, it may be preferable to simply re-sequence individuals when relevant, thereby removing many of the concerns of storing the data (albeit this approach would also remove, to some extent, the suggested advantage(s) of having the sequence ‘on hand' to serve in the larger framework of genomic or personalized medicine in the future). Moreover, respect of newborns' right to privacy, right not to know and autonomy to give consent once they are of legal age also suggests that storing the whole-genome sequence information for further testing in childhood is premature. Nor is storage without potential risk to privacy and confidentiality; it is becoming increasingly evident that fully guaranteeing the anonymity of WGS data may be impossible.^30^ Policy makers need to consider not only the information content of the genome but also its role as a biometric that can be used to identify and track individuals, and their relatives. Furthermore, the costs of storing the data are unknown and may be high.

An additional relevant phenomenon when discussing the sequencing of large portions of the genome, including WGS and WES, is that of unsolicited findings. These are findings or results that are not directly related to the scope of the initial research or clinical question(s). Many different terms have been used to describe such findings, including incidental findings, unanticipated findings and off-target results.^31^ Despite the different (and potentially confusing) terms, they are often used in this context to refer to unexpected findings that are in some way ‘stumbled upon' and/or a ‘surprise' to the researcher or clinician, unrelated to the initial reason for sequencing, and as such raise questions about whether they should be returned to patients or research participants. Unsolicited findings are not a phenomenon restricted to genomics, they arise in clinical practice (eg, imaging) and in other forms of biomedical research. They have, however, become a focal point of debate in genomics owing to the fact that we can now generate such large quantities of DNA almost all at once and thus have access to much more data regardless of whether they are related to the initial research or clinical question posed. In order to reduce the chances of encountering unsolicited findings, authors suggest targeting the sequencing and/or the DNA analysis to regions of the genome that are directly relevant to the research question.^32^ Although this approach will, indeed, help to avoid unsolicited findings, they cannot exclude them entirely.

Even with these present challenges, the potential reality of using NGS and WGS in NBS is drawing closer as revealed by the recent funding (25 million USD over 5 years) by the National Institutes of Health in the United States of America of various pilot projects that will study the implications, challenges and opportunities associated with the possible integration of genome sequencing in newborns.^33^ These projects aim to study different ways in which genome or exome sequencing can be implemented in populations of newborns, for example, one project will offer genome sequencing to asymptomatic newborns, whereas another project aims to offer genome sequencing to newborns who have symptoms and are being cared for in the neonatal intensive care unit.^34^ These research projects may further raise expectations about the use of high-throughput sequencing technologies and genome sequencing in NBS. Now is the time to consider what may be appropriate and anticipate developments while closely monitoring developments in order to determine and clearly describe whether and how genome sequencing may be used for newborn populations. As indicated by the different approaches used in these pilot projects, and keeping in mind that having access to new tools that offer more possibilities, does not bind us to the imperative of having to perform all that is possible simply because we can, it will be essential for health-care systems to determine and clearly describe whether and how NGS-based or genome sequencing is to be used for newborn populations: should genome sequencing be used in NBS programmes? If so, is it expected that genome sequencing will be used as a first tier testing programme and as such will all babies, automatically, (and by default) have specific regions or their whole-genome sequenced and/or analyzed at birth?

Another use of genome sequencing in newborns would be for the diagnosis of sick babies, however, as this does not fall under NBS and is ethically distinct from such programmes, it is beyond the scope of this paper. Nevertheless, a similar dichotomy of approaches (screening healthy children versus diagnosis of symptomatic children) has been described by Wade *et al*^35^ regarding the use of WGS in pediatric populations. Clearly, the approach chosen will depend on the determined goal of the programme, and will have an important impact on the resulting practical and ethical issues, including the benefits and disadvantages of such a programme. As stated by Wade *et al*,^35^ ‘Like most clinical tools, P-WGS (Pediatric whole-genome sequencing) could be used for a range of purposes... Therefore, meaningful assessment of P-WGS can be accomplished only when a clear health care context has been specified, including the population target and purpose of testing'.^35^ It should also be noted that screening the entire population of newborns would not necessarily dictate the number of conditions that would be studied; it could be decided to study the same number of conditions presently offered at birth or it could be decided to expand the panel of diseases even further. This decision is obviously also very important and the debate over the criteria or reasons to expand would, at present, to some extent, echo the ongoing debate over expansion of NBS in the last decade, and we do not offer herein a solution or recommendation regarding this debate. Once the general approach of how to implement genome sequencing in NBS is determined, programmes will still need to define which conditions will be screened for and/or which results will be returned.^36^ We stress that this discussion regarding which conditions and which variants to include in the screening panel should be discussed with all relevant stakeholders, including clinicians, researchers, ethicists, public health professionals, policy makers and patients (or patient representatives).

## NBS and genome sequencing: which way forward?

Should genome sequencing be used for NBS, a major expansion of health information (including validated and non-validated, highly or poorly predictive, more or less probabilistic and affecting mainly adult-onset or childhood-onset conditions) and non-medical information from an analysis of the sequence data might be generated depending on how the sequence is analyzed. Despite the scientific and public enthusiasm toward analyzing this wealth of information in diagnostics, research and screening, this reality demands careful appraisal with regard to the justification for using new sequencing technologies and genome sequencing approaches, and the proportionality of using them.^37^

### Genome sequencing and NBS: a focused screening approach

Neonatal screening programmes have traditionally focused on disorders with serious outcomes having accepted treatments, recognizable latent or early symptomatic stages, a well-understood natural history, a suitable test and an acceptable cost-benefit evaluation. It could be possible to use new sequencing technologies as tools in NBS without fundamentally changing this model. However, it is obvious that the use of such tools (and any consequent sequencing approaches, including WGS) has the potential to cause a paradigm shift in NBS. As mentioned above, obtaining data on, and studying a restricted number of genes and variants is a very different scenario than generating data and studying the entire genome. With the present capacity of producing a greater amount of data, it is essential that we carefully consider our stance on the further expansion of the NBS panel and therefore the discussion of the diseases to be included in such a panel.

In general, for most common complex disorders, the genetic variants that are currently understood constitute a relatively small etiologic factor. Little evidence exists supporting the notion that the use of WGS for common complex disorders will result in clinically actionable information other than general health advice urging for a healthy balanced diet, doing physical activity regularly and, in general, abandoning unhealthy behavior.^38^ Although the focus on common complex disorders in seeking to apply new genetic technologies to public health is understandable, more might be gained by focusing on rare disorders. Many individuals carry rare variants that might provide preventive advantages if knowledge about their genetic risk was available.

Therefore, in line with the original intention of NBS, the potential application of new sequence technologies and genome sequence approaches should have as their primary objective to focus on the identification of highly penetrant disease-causing variants that confer a high risk of preventable or treatable conditions during the newborn and childhood period. As a consequence, this would include the selection of variants and genes that clearly cause disease and are known to have a high penetrance with effective and accepted preventive or therapeutic interventions.^39^ This approach would thus include the sequencing of targeted genes on a panel instead of sequencing or analyzing the whole genome. In line with the view of the International Society of Neonatal Screening, ‘screening tests should not be recommended if indications of advantage from early diagnosis are lacking or uncertain, or the test is unsuitable, or does not detect those cases in which there might be an advantage'.^40^

Indeed, at the very least, the conditions included and returned to parents at birth should offer clinical benefit in the near term, that is to say during the newborn and childhood period. As stated by Caulfield *et al*^41^ regarding the use of WGS in the clinic ‘Utility in a clinical setting depends on many—and very different—factors, and must take into account not only such performance characteristics as sensitivity, specificity and positive and negative predictive value, but also demonstration of beneficial impact of using the test on patients' health, or on health services delivery. Failure to do so can trigger overt harm to patients in addition to excessive cost to the healthcare system'.^41^

### Genome sequencing and NBS: evidence generation and responsible cost appraisal

It still has to be proven whether the implementation of new sequencing technologies and approaches in NBS would be an effective public health strategy. Even if used in a targeted fashion, evidence will have to be generated with regard to the development of a suitable test (including the identification of the targeted variants), the targeted conditions, the development of a calculation of costs and consequent actual analysis of costs, the treatment pathways, the potential impact on test recipients and the acceptability to the population. Moreover, various other ethical, legal and social issues will also have to be addressed before introduction.

Admittedly, in economic terms, the use of NGS as a screening tool will only be cost effective when a certain minimum number of genetic variants or genes are to be included. Also of note is the fact that if the sequence generated at birth is to be used for further medical enquiries throughout the individual's lifetime, the economic evaluation of the cost and benefit of using this technology and consequent sequencing approaches becomes much more complicated than simply analyzing the costs and benefits potentially incurred at birth and in early childhood. The costs of storage of the data, its future or ongoing interpretation, as well as the costs for follow-up or confirmatory testing should be considered. Additional downstream costs to the health-care system should also be considered. As explained by Andermann,^19^ ‘Although genetic services and screening programmes aim to improve the health of the population, there is growing concern that the increasing number of genetic tests becoming available at lower costs could compromise the viability of the healthcare system. Even though the tests themselves may be inexpensive and suitable for large-scale use, the infrastructure and human resources needed to provide appropriate education, counseling, interventions and follow-up are likely to be far more costly. When it comes to the allocation of scarce resources, economic considerations must be considered alongside ‘notions of justice, equity, personal freedom, political feasibility, and the constraints of current law'.^19^

### Genome sequencing and NBS: focus on the best interests of the child

Debates on the remit of NBS must also confront differing interpretations of what constitutes the ‘benefits' of NBS: from what is good for the infant to what might be potentially good for the infant, to what might be good for the family (eg, reproductive benefit or health benefits for family members) or to what, currently, might be beneficial for society at large (eg, for research).^42^

Although NBS might indeed lead to the identification of information that is relevant for the parents (eg, carrier status), the primary justification of doing neonatal screening should be the health interests of the child. From this perspective, the goal should not be in the purposeful identification of information beyond the primary goal of the screening. However, if the detection of an unsolicited genetic variant would be indicative of serious health problems in the minor that allows for treatment or prevention, a health-care professional should report such variants.^37^ Furthermore, parents may also be offered information regarding unsolicited findings that are severe and clinically actionable relevant to their own health. This occasion will likely arise rarely as laboratories will rarely find or ‘stumble upon' additional predisposing variants in the process of analyzing the genes targeted by NBS programmes. The authors strongly recommend that laboratories should not examine genes beyond those targeted in screening.

Any approach to screen newborns using genome sequencing should be coherent with general principles regarding genetic testing in children. ‘There has been a longstanding consensus that the primary and strongest justification for genetic testing of children exists when the results will clarify the cause of current symptoms, when the onset of the condition may occur during childhood, or when the information will be used to embark on a course of care that must start during childhood to prevent or ameliorate later symptoms'.^43^ A major rationale behind this position is that testing in children should be delayed until the person is old enough to make an informed choice. In clinical care, this reflects the careful consideration that is currently given through pretest genetic counseling, where special attention is paid to the transfer of information about the test and the test results, the confidentiality of genetic information, the voluntariness of the request, the responsibility toward blood relatives and the psychological impact of a test.

### Genome sequencing and NBS: informed consent

NBS is usually conducted without explicit consent because it is considered to be in the best interest of the child's health and, as a consequence, part of routine pediatric care for newborns. Various studies reported that many parents experienced NBS as a largely routine practice and felt uninformed about the procedure.^44^ In reaction to this, efforts were made to inform parents about the disorders screened and the potential consequences of the screening.^45^ Most consider this model of presumed consent to be an appropriate approach in the context of NBS for conditions that provide clear evidence of medical benefit for the newborns. Concerns have been raised that a model of explicit consent could lead to a reduced uptake in screening,^46^ although there is little evidence to support this. If genome sequencing is used in the context of NBS, new models of informed consent in the context of NBS will have to be developed that on the one hand increase the information provided and ensure it is provided at the right time,^47^ and on the other hand maximize participation rates, as the main focus of NBS should be finding the at-risk asymptomatic child for whom prevention or treatment is available during childhood. Furthermore, another important consideration is the amount of time a health-care professional will need to communicate the necessary information and obtain informed consent. The potential need for (more) time and resources for consent should also be considered in light of the suggestion that before testing, parents be made aware of the possibility of generating unsolicited finding.

### Genome sequencing and NBS: storage of sequence data and samples for research

Samples from NBS are usually stored in public health laboratories in order to permit diagnosis, re-testing to confirm results, postmortem diagnosis, and for laboratory audit and quality control. The short-term storage of these samples for these purposes is generally not particularly controversial, as these uses are related to the primary purpose of the initial collection. However, the issue of storage of NBS samples for a longer term has become an important matter of debate.^48^ The potential generation of sequencing data in the NBS context and the storage of residual NBS biological material would clearly provide opportunities for research that might contribute significantly to health. However, given the particular sensitivity of the governmental context of NBS programmes, such storage would only be possible with well elaborated explicit consent procedures and adequate privacy and confidentiality procedures.

### Genome sequencing and NBS: use of sequence data in the clinical file

Careful considerations will have to be made about who would undertake and be responsible for the potential further analysis of this sequence data and deciding what will be kept in the medical record. The need for better education and training of health-care professionals has been raised as an important goal as we prepare for a health-care setting where genomic medicine is expected to have an increasingly larger role. The question about whether clinicians will be able to interpret the copious amounts of data generated by genome sequencing, including WGS and WES,^6^ and how we can ensure that they are properly trained to do so is of fundamental importance.^49^ This will become more and more important if genome sequencing is to be used in the newborn period and if sequence data is to become part of the medical file (even if some parts would not have been analyzed at birth, but would be used at specific moments in time, for instance, at reproductive age).^28^ A clear protocol for the safe storage of the data in electronic medical files should be elaborated. The analyzed results as well as unanalyzed data should be handled and treated like all clinical information included in patients' medical file, and be protected by adequate privacy and confidentiality procedures.

### Genome sequencing and NBS outside public health programmes

A relevant point to keep in mind in the discussion of using genome sequencing in newborns is the fact that private companies outside of the public health-care system are currently offering WGS to consumers. As such, this could be a way for parents to have their child's DNA sequenced at any stage of life, regardless of what the traditional public health-care screening context offers.^50^ As with the ongoing discussion about genetic testing offered direct-to-consumer, this fact raises the question of whether any other form of regulation and/or guidance may be needed for genetic services offered outside the traditional health-care system.^51^ As commercial offers of genome sequencing might affect the public health-care system and various ethical concerns are similar whether genome sequencing is happening in a private setting or as part of a public health context, the reflections and recommendations developed in this document apply in both settings.

## Conclusion

For more than four decades, NBS programmes have been set up worldwide in order to identify infants for whom early treatment or preventive actions would provide a clear health benefit to the child. The discussion regarding the use of next-generation technology as a tool or the use of genome sequencing approaches in NBS raises questions about a possible paradigm shift in health care: will we use new sequencing technologies as a tool to answer focused clinical questions or will we use it to sequence entire genomes in order to return a set of results at birth and as a pure data and information generator, much of which can be analyzed and returned throughout a person's lifetime? The first approach raises differences in scale but is amenable to our current forms of analysis such as cost effectiveness and evaluation of population health programmes and so on. The second approach is partially based on an assumption that personalized medicine based on analysis of the genome is a potential reality, desirable and an effective use of scarce health-care resources.

The availability of NGS technologies creates high expectations regarding the potential of these technologies in the context of NBS. However, the potential implementation of genome-sequencing technologies in NBS will take years and will necessitate further societal and professional debate on the desired aims of neonatal screening with a wide variety of stakeholders involved.^36^ Indeed, there is still much to be learned in terms of science as well as the ethical, legal and social issues regarding the use of new sequencing technologies and genome sequencing approaches in health-care and public health programmes; we should avoid hasty action and untimely application of these tools and approaches in NBS programmes.

It should be noted that during the public consultation process in the preparation of this document, some NBS and/or genome sequencing-related issues were raised by various stakeholders that merit further attention but unfortunately extend beyond the scope of this article. These include the discussion of the role that policy makers may have in considering the implications of integrating genome sequencing into NBS as well as more fundamental issues related to the public (mis)understanding of the role of genetics in determining health status, impact on insurability, the discussion of genomic sequencing in NBS within the wider context of genomic sequencing for prenatal (noninvasive) screening or preconceptual carrier screening, and how such screening programmes may impact upon society's view and understanding of health, and the potential inequalities between countries that can afford to use genomic technologies and those that cannot. Although we cannot address all of these issues herein, we hope that this document will help initiate further discussion and research regarding these important issues.

Finally, the recommendations proposed in this statement provide a first orientation in the debate on the potential implementation of genome sequencing in NBS.

## Recommendations

NGS technologies are powerful tools that allow for novel approaches for studying the genome, including generating the entire nucleotide sequence of an individual. With respect to this capacity to study DNA variants in relation to disease, it can be used at different stages of life, including at birth. The following recommendations are not meant as an encouragement to use new sequencing tools or genome sequencing approaches in NBS programmes, but rather they offer a list of areas that must be carefully addressed should stakeholders consider using said technologies and approaches in NBS programmes. Furthermore, before such tools and approaches are introduced into NBS programmes, the goals and values of these programmes should be explicitly articulated. The responsible use of genome sequencing within a public health programme such as NBS should not be technology driven, but rather be adopted on the basis of its public health potential. The primary justification for performing genome sequencing within the context of NBS should be the health interests of the child. We recommend that the following issues be addressed when considering whether genome sequencing be incorporated into NBS programmes.

### Purpose

The primary objective of genome sequencing in NBS should be the identification of gene variants conferring a high risk of preventable or treatable conditions, for which treatment has to start in the newborn period or in early childhood. This includes the selection of variants that clearly cause disease, are known to have high penetrance, and for which effective and accepted preventive therapeutic interventions are available. At this time, we recommend a targeted sequencing or targeted analysis approach.

### Evidence

A robust evidence base is a prerequisite for responsible and effective NBS. This evidence base requires the understanding of the presence of variants in certain populations, the sensitivity and specificity of tests, the identification of treatment pathways or preventive action, the calculation of immediate and downstream costs, the assessment of lives saved, quality of life gained and the potential impact on families, and, finally, the determination of public acceptability.

### Costs

The infrastructure and human resource costs to ensure monitoring, appropriate education, counseling, interventions and storage must be assessed. Appropriate studies should be undertaken to obtain a realistic picture of all costs involved as part of NBS in a public health programme, including subsequent diagnostic costs as well as the costs of not implementing genome sequencing.

### Engagement

The perception of the harm and benefits of screening is very different among different stakeholders. For this reason, it is important to have an open dialogue about the expected medical and social benefits with all stakeholders, including patients and their representatives, and the general public. Moreover, governments should engage with key opinion leaders to establish sound policy to guide any integration of genome sequencing into NBS. This would enable a population health approach to be followed, key frameworks to be developed, support decision making in relation to the conditions for which screening could be offered and would support a consistent approach between jurisdictions within a region.

### Informing parents

Even if only targeted genome sequencing is adopted, new NBS models of informing parents will have to be developed. They should provide the necessary information but also maximize participation rates as the main focus of NBS should always be the identification and treatment of the asymptomatic, at-risk newborn. These should also include information about the possibility of finding unsolicited results, potential storage and research uses of the samples and data.

### Educating professionals and the public

Attention should be paid to the particular education and training needs of the health-care professionals involved in NBS. This includes the development of public education programmes to increase public understanding of both genomics and NBS, their benefits and limitations.

### Future uses beyond NBS

Storing the whole-genome sequence or large amounts of sequence information of newborns for health-care purposes is premature at this time. Policy makers need to consider uses to improve public health and research, as well as their potential to identify individuals and their relatives.

### Unsolicited findings

An approach using a targeted sequencing or targeted analysis will likely limit the number of unsolicited findings. However, unsolicited findings indicative of serious health problems for the child should still be reported to parents where treatment or prevention is available during childhood. Moreover, as carrier status information may be relevant to parents for reproductive choices, they could be offered this information. Parents may also be offered information regarding unsolicited findings that are severe and clinically actionable relevant to their own health.
